# A Few Points in the Treatment of Pneumonia

**Published:** 1854-03

**Authors:** G. William Semple

**Affiliations:** Hampton, Va.


### *A few Points in the Treatment of Pneumonia.*

BY G. WILLIAM SEMPLE, M. D., HAMPTON, VA.

In the April number for 1853, of the Virginia Medical and Surgical Journal, is published an excellent article on pneumonia, by Dr. Thos. Johnson. The author presents, in a succinct, but clear and accurate account of the symptoms, anatomical character, and physical signs of inflammation of the pulmonary parenchyma, a good portrait of that disease. The treatment proposed is well stated; I fully concur in all that is said of the efficacy of general blood-letting, the contra-stimulant effect of tartar emetic, and the antiphlogistic and other effects of calomel.

It has been my practice, after administering a full purgative dose of calomel in the commencement of an attack of pneumonia, to continue its exhibition during the first and second stages of the disease, whilst employing contra-stimulants and venesection, in such doses as to procure one or two free alvine evacuations in the course of every twenty-four hours, with the further view of lightening the office of the lungs, by the decarbonization of the blood through means of the bilious secretion thus produced, and also of placing the system in a condition for the more ready and rapid induction of the constitutional effects of mercury, should such a course be afterwards deemed necessary. On the speedy production of these effects in many urgent cases, the lives of patients frequently depend.

It has been my fortune to meet with not a few cases of violent pneumonia, involving the greater portion of one, and sometimes extending to both lungs, which, after resisting every effort to reduce them without the slightest abatement or reduction of the extent of the inflammation, have been suddenly relieved by the spontaneous occurrence of a critical diuresis, when the condition of the patients seemed almost hopeless. This fact suggested to me the addition of a few drops of tincture of digitalis to each dose of the solution of tartar emetic, both with the view of inducing a critical diuresis and of aiding the sedative effect of the antimony; the successful results of this method have given me abundant reason to continue to employ it.

"I do not believe that such a thing as intermittent pneumonic inflammation ever exists," but whatever debilitates, in a miasmatic region, favors the development of intermittent fever. Thus one who has had a copious epistaxis or other hæmorrhage, or has suffered from cholera morbus or an exhausting diarrhœa, goes out on a damp chilly day in autumn or winter and returns with an ague. The slight exposure enabling the latent poison of the intermittent to develope itself in a debilitated constitution, though it might never have done so whilst the patient continued unreduced. The debilitating treatment necessary to reduce a pneumonia in like manner favors the development of intermittent fever, and accordingly, in miasmatic regions, it often makes its appearance when the inflammation begins to decline. There are few physicians practicing in the tidewater country of this State who will not concur in these views; and scarcely one, who, on visiting some patient suffering from pneumonia, from the fifth to the eighth day, whom he had left on the day before in an improved condition, has not been told that on the preceding evening he had suddenly become much worse, had had a rise of fever, much thirst, increased frequency and difficulty in respiration, pain in the side, headache, pain in the loins and lower extremities, and perhaps some delirium; but that in the course of some hours a copious perspiration, attended by a free discharge of urine, had come on, since which he had again become better, though still not so well as on the day before. On examination he has found that dulness on percussion and bronchial respiration have extended, and that crepitation is heard over a greater extent of surface, though the pulse is softer, slower, and less frequent, and the skin is moist or even freely perspiring.—When such a case occurs, if the physician do not recognise from the symptoms a paroxysm of intermittent fever, but concludes that from some unknown or accidental cause there has been only an increase of the pneumonic inflammation which is now perhaps in the course of being relieved by the occurrence of a favorable crisis, and prescribes accordingly; if the intermittent now complicating the pneumonia be of the quotidian form; on visiting the patient the next day he will find that the same train of symptoms have recurred. But now the inflammation has perhaps extended so far that the patient, bathed in a copious perspiration, is gasping for breath. Woe to his patient, if he do not now recognise the intermittent before the third paroxysm. No treatment adapted only to the cure of pneumonia can possibly avail Quinine and quinine only, in full doses, can arrest such paroxysms and leave room for a reduction of the inflammation by subsequent treatment.

Such attacks occurring in the course of pneumonia may assume the quotidian, tertian, double tertian, or any other type of intermittent fever. Whatever be the type, if the patient be closely attended, and the symptoms and physical signs attentively observed, it will be found from the latter, as might be anticipated, that the inflammation regularly and progressively extends during the cold (which is usually very short) and hot stage, and that a regular diminution of the inflammation succeeds, during the sweating stage, caused by the critical evacuations which then occur, though a larger portion of the lung or lungs is left impaired or spoiled after each successive paroxysm.

So common has the complication of pneumonia with intermittent fever become, that many of my medical acquaintances have adopted, as well as myself, the practice of exhibiting quinine in doses of from three to four grains, every third or fourth hour, as soon as the physical signs indicate a diminution of the extent of the inflammation.—The expectoration usually improves, and the respiration becomes less laborious.

As intermittent fever frequently complicates pneumonia, so also does pneumouia often complicate vernal intermittent fever. In the spring of 1850, scarcely a case of intermittent fever occurred in this neighborhood which was suffered to progress beyond the second paroxysm, that was not complicated by the occurrence of pneumonia. The arrest of the intermittent did not cure the pneumonia, but it required subsequent treatment, and sometimes even proved fatal.—*Vir. Med. and Surg. Jour.*

